# The Effectiveness of Interventions to Increase Participation and Physical Activities in Parks: A Systematic Review of the Literature

**DOI:** 10.3390/ijerph191912590

**Published:** 2022-10-02

**Authors:** Ying Xu, Sarah Ann Wheeler, Alec Zuo

**Affiliations:** 1 School of Economics and Public Policy, The University of Adelaide, Adelaide, SA 5005, Australia; 2 The Centre for Global Food and Resources, School of Economics and Public Policy, The University of Adelaide, Adelaide, SA 5005, Australia

**Keywords:** park use, physical activities, interventions, risk of bias, quality of evidence

## Abstract

Although a variety of interventions in many countries have been developed to increase park use and to improve public participation in physical activities in parks, knowledge of their overall effectiveness is lacking. A systematic literature review was undertaken to further understand the effectiveness of various interventions that aim to increase the use of parks and physical activity participation in parks. This systematic review utilized the standard Preferred Reporting Items for Systematic Reviews and Meta-Analysis procedure, and of the 3801 studies identified, 60 studies were reviewed in-depth, with 44 studies assessed for quality of evidence and risk of bias. Most of the 44 reviewed studies found that various interventions had positive impacts on park use and physical activity participation in parks. Interventions were classified into both demand and supply, with more studies focusing on the supply side. The strongest evidence on the effectiveness of various interventions was found for park prescriptions; safe access to parks; playgrounds, and park renovation and renewal/design. However, the assessment of quality of evidence and risk of bias showed that most studies suffer from potential biases and evidence weaknesses, suggesting a need to further establish external validity.

## 1. Introduction

Physical inactivity is a growing trend in many countries. Scholarly studies have associated the lack of physical activity with several leading causes of death, including cancer, stroke, diabetes, and heart disease [1]. Although physical activity can generate numerous health benefits, many adults fail to achieve the recommended level of physical activity across countries [2]. From a public health policy perspective, how to scientifically design and to implement certain interventions to improve public participation in physical activities in a cost-effective manner remains an open question that needs rigorous investigation.

In many countries, governments at different levels have broadly considered parks as a major venue of stimulating physical activities among the public. Park and its impact on physical activity are associated with walkability and mobility factors [3]. Additionally, many studies show that parks are positively linked to mental health-related outcomes [4,5,6]. For example, Sturm and Cohen [4] found that mental health is significantly related to residential distance from the park, while the highest mental health score is found among residents within short walking distance from the park. Orstad et al. [6] found that park proximity was indirectly associated with fewer days of poor mental health, mediated by park-based physical activities. It is generally believed that parks can provide a wide range of low-cost or free physical activities, many of which can be exercised in proximity to people’s residences [7].

There is a growing trend of studies investigating potential determinants of physical activity participation through park visits, with findings on the effects that numerous factors have on physical activities in parks. In these studies, the factors that affect physical activities in parks can be categorized into three groups: (1) park users (at both individual and neighbourhood levels), (2) park attributes, and (3) park proximity. At the individual park user level, these include sociodemographic characteristics such as gender and age [8,9,10,11,12]; household characteristics such as family structure [13]; and neighbourhood features such as land use mix, street audits, level of deprivation, neighbourhood walkability, crime rates, and perceived park safety by the residents [14,15,16]. In addition, factors such as people’s willingness to pay for higher biodiversity or lower numbers of visitors are also found to play a role [17,18]. It is further found that people’s park visit decisions are also inter-related with their other neighbourhood attributes such as road safety, aesthetics, and facilities [15,19].

At the park level, several attributes have been found to be important in influencing people’s physical activities in parks: park amenities [20], capacity [21], provision of advanced services [22], and availability of recreational facilities [23], especially multigenerational playgrounds with access to various activities and fitness zones [24].

Park proximity includes proximity to recreational facilities in parks [25] or proximity of parks to an individual’s home. It includes the availability of parks within a short distance from a residence, which usually stimulates physical activities at these places [26,27]. There are different ways to measure the proximity of parks based on park attributes. For example, Ou et al. [26] developed three proximity measures. The first measured the distance between participants’ homes and the nearest park. The second measured the distance to the nearest park with sports/walking facilities. The third measured the distance to the park that participants perceived as usable for physical activity.

The above-identified three determinants (user, park attributes, and proximity) of physical activity participation in parks can be grouped into two categories: (1) demand-side factors (individual sociodemographic, economic, and psychological characteristics); and (2) supply-side factors (park provision, access, and attributes). Understanding these influences is important given the need for policies to be effective and efficient in stimulating physical activities in parks.

Interventions in parks usually aim to change healthy behaviours by either stimulating demand, increasing supply, or reducing barriers to park visits and physical activities [28,29]. Recent literature reviews have focused on environmental interventions to encourage the use of green space [30], park-based physical activities [31], and general physical activities [32]. Several other literature reviews examine the effects of urban green space interventions on physical activities, or other health, wellbeing, social, environmental outcomes [33,34]. Audrey and Batista-Ferrer [35] explored how interventions could change urban environment and health outcomes.

Most of these reviews focus on one or two outcomes such as encourage of park use, physical activities, or mental health outcomes. To the best of our knowledge, Derose et al. [36] was the first systematic review that examines park-based interventions on health-related outcomes, while Wallace et al. [29] provided a systematic review protocol on the effects of park-based interventions on health. However, the examination of Derose et al. [36] is based only on two forms of interventions, which are place- and person-based, and 27 studies were included in the review. Knowledge gaps still exist regarding the possible effectiveness of more interventions that are in place or have yet to be trialled and evaluated, which perhaps can only be synthesized from a larger number of studies. Additionally, broader effects on all aspects of park uses, physical activities, and health-related outcomes need to be examined. Furthermore, few studies have rigorously assessed both quality of evidence and potential risk of bias, which need to be clearly understood to help establish external validity.

This study seeks to narrow these gaps through a comprehensive systematic literature review. It identified and examined 17 park interventions and their respective effects on ten outcomes including park use, physical activity participation, and health-related measures. In doing this, it applies a rigorous literature synthesis protocol and presents a more complete list of existing studies in this regard. It further evaluates the quality of evidence and risk of bias to assist the design and implementation of future interventions. The study results will be useful to environmental scientists, public health professionals, and policy-makers in better utilizing their resources in developing interventions to achieve goals of increasing participation in physical activity in parks.

## 2. Methods

### 2.1. Peer-Reviewed Literature

This review is based on published literature that only reports findings at aggregate levels where no individual information is meant to or possible to be identified and thus is exempt from ethical compliance. It followed the standard Preferred Reporting Items for Systematic Reviews and Meta-Analysis (PRISMA), namely (1) identification of literature, (2) screening question, and (3) eligibility using inclusion criteria. We conducted a two-stage process to identify the relevant studies. First, we undertook a search of literature in electronic databases PubMed, Web of Science, Scopus, and CINAHL published to date. We then used a combination of keywords to search the literature related to (1) park, (2) physical activity, and (3) intervention (Table S1 in the Supplementary Materials).

This stage of search returned 3801 studies. One reviewer (Y.X.) screened the titles and abstracts to identify those that were eligible for the systematic review, according to the following inclusion criteria:

(a) In English;
(b) A peer-reviewed and published journal article;
(c) Full text available for review;
(d) Relevant to park visitation or physical activities in parks.

**Figure 1:**
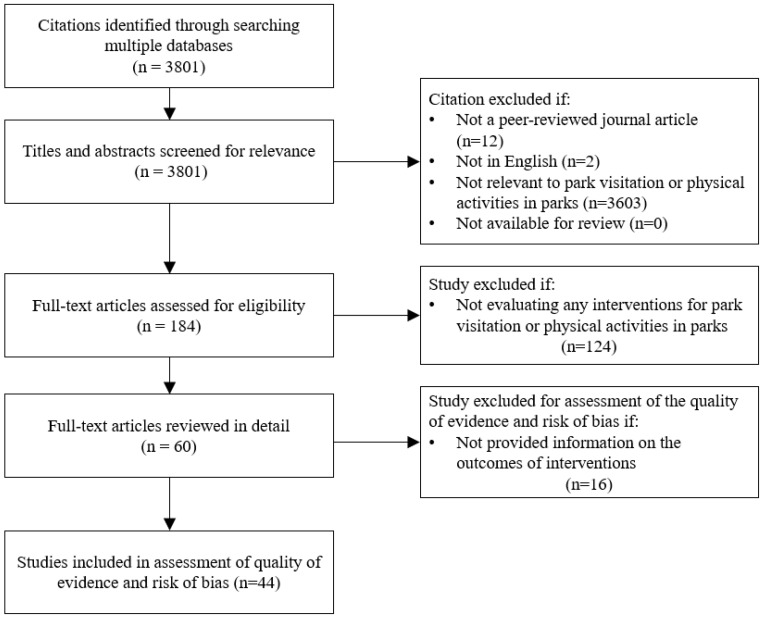
PRISMA flowchart of the literature selection process. Source: Authors’ own design.

The definition of parks includes national parks (conservation, recreation, wilderness, and regional reserve); reservoirs; botanical gardens; forestry reserves; and state, county, city and neighbourhood parks. Two studies were excluded because they were not published in English. Another 12 articles were excluded because they were not peer-reviewed or published journal articles. Most studies (n = 3603) were irrelevant to park visitation or physical activities in parks and, therefore, were excluded. However, the remainder (n = 184) were available for review. After the initial screening, these 184 studies were identified from subscription only and open access publishers. In the second stage, we reviewed these studies, with 60 full-text articles determined to be relevant as interventions to facilitate participation and physical activity in parks, among which 44 studies provided information on the outcomes of the interventions. Hence, 60 selected articles were used for our basic analysis (e.g., geographical coverage, study design, and population of interest), but only 44 studies were used for evaluating the impacts of interventions and assessing the quality of studies. A flowchart of the literature selection process is provided in Figure 1. The selected full-text studies were then screened by all three authors to abstract information and to assess evidence. The 60 selected studies were reviewed in detail, and comprehensive information was abstracted, including published year, country, population of interest, type of study design, and type of interventions. The findings, particularly the significance and magnitude of impacts of interventions on park uses, physical activities, and health outcomes, were abstracted by reviewing 44 studies. The quality of evidence and risk of bias were also evaluated using 44 studies.

### 2.2. Evidence Grading

Quality of evidence was assessed using the Grading of Recommendations, Assessment, Development and Evaluation (GRADE) approach [37]. This approach is a standardized way to rate the quality of evidence of clinical and public health studies. A full description of the GRADE rating system was described in Balshem et al. [38]. The GRADE rating system considers five domains, including risk of bias, consistency of results, indirectness, imprecision, and publication bias for assessment. Each domain needs to be scored to obtain an overall evaluation outcome. The evaluation outcomes for applicable studies were assigned one of four ratings (1 = very low, 2 = low, 3 = moderate, and 4 = high) to indicate the quality of the evidence obtained from the relevant studies. To conduct rating, we considered randomization, allocation concealment, blinding, use of intention to treat analysis, as well as appropriate control of confounding [37].

Risk of bias was assessed using the Cochrane Risk of Bias assessment tool for Non-Randomized Studies of Interventions (ACROBAT-NRSI) [39]. Though it was developed primarily for randomized control trials (RCTs), it was also widely used for non-randomized studies as the criteria are also applicable for assessing the weakness present in non-randomized studies [30]. It includes seven domains of bias (e.g., bias due to confounding, bias in selection of participants into the study, bias in measurement of interventions, bias due to departures from intended interventions, bias due to missing data, bias in measurement of outcomes, and bias in selection of the reported result). Each domain contains the signalling questions providing an evidential basis for risk of bias assessment (Table S3 in the Supplementary Materials). To conduct assessments, each domain needs to be judged to obtain the overall risk of bias judgment. The different levels of judgments of each domain include low, moderate, serious, and critical. The judgment of the overall risk of bias is based on the judgment of each domain. According to ACROBAT-NRSI, the overall risk of bias at least equals the most severe one among the seven domains. Additionally, if there are four or more domains of the same severe level of risk of bias, the overall risk of bias will be rated to be more severe [30,39].

This review provides a thorough appraisal of GRADE and the risk of bias in the experimental studies. Estimating the quality of evidence through these is necessary because various qualities lead to different validity and reliability of results while assessing the impacts of interventions on park uses and physical activities. Therefore, the appraisal of the quality of evidence could mitigate the effect of biases and assist in providing the findings based on high-quality studies. The eligible studies had to be evaluated for the impacts of interventions, including studies with at least one measurement of park uses/physical activity before and after the intervention, or studies with at least one measurement of park uses/physical activity that compares the treatment group with control group.

## 3. Results

### 3.1. An Overview of Systematic Review Publications

**Figure 2:**
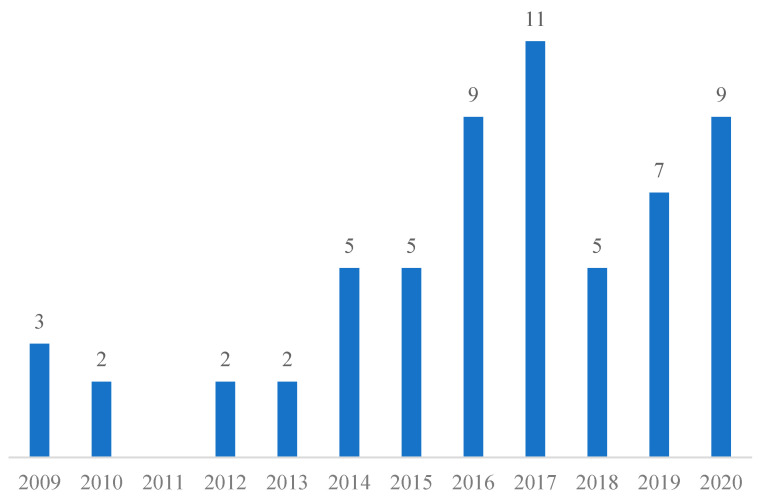
Annual numbers of systematic review publications. Source: Authors’ own multi-database literature search.

How public interventions can stimulate people’s participation in park-based physical activities is an emerging issue, with the first study published only a decade ago. The 60 selected closely related peer-reviewed studies were published between 2009 and 2020. The annual number of publications shows an overall increasing trend over time (Figure 2).

These publications covered a total of 13 countries. As a few studies focused on more than one country, the total number of investigations came to 64 (Table 1). Most studies focused on developed countries, with only two studies from Colombia, the only lower-middle-income country on the list. The coverage was disproportionate to either land area or population, suggesting that the existing knowledge is therefore not globally representative and has been a topic that has only been of interest in higher income countries. In particular, the United States had over half of the studies identified, followed by Australia with 16 studies and then Canada.

### 3.2. Study Design

Table 2 reports the study design of selected publications (n = 60). The majority of publications (68%) used quasi-experimental evaluation designs with or without a comparison group. No matter with or without a comparison group, these quasi-experimental designs did not randomly assign participants to treatment and control groups. Though it is easier to implement compared to a randomised controlled trial, quasi-experimental designs have poor internal validity, which is the ability to assert that an intervention has caused an outcome. Randomised controlled trials, usually regarded as the best method to test the effectiveness of new treatments by reducing certain sources of bias, accounted for 20% of all the selected studies. Moreover, there are also a few case studies and several systematic reviews.

### 3.3. Population of Interest

The population of interest in these studies is characterized in Table 3. Almost half of publications (28) focused on all age groups. Other than these, six focused specifically on children and seven focused on adults. The studies focused on children mainly explore the interventions of park renovation, especially playground renovation or installation [40,41,42]. Park-prescription intervention [43] and non-physical activity programs were also investigated [44]. In contrast, intervention types for adults are more diverse than those for children, including physical activity programs [45], outdoor gym installation [46,47], redevelopment of parks [48], dog parks, as well as park prescriptions by medical professionals [49,50].

Although the older population faces a series of challenges such as social isolation and a lack of exercise, only two studies specifically focused on them. Gagliardi et al. [51] explored the socializing and outdoor activities for older people in parks and found the significant effect of these programs in promoting older people’s physical activity. However, Cohen et al. [52] found that renovation to the senior centre by adding a bank of exercise machines along with fewer programmed activities results in less participation of older people. This study indicated that physical features might be less important than social factors in attracting older people to parks.

### 3.4. Type of Interventions

Multiple interventions were evaluated in the selected literature. These interventions were implemented from either the supply side (provision of parks and access) or the demand side (stimulation of public participation in park visits and physical activities). Specifically, the 12 supply-side interventions covered in this study included (numbered for later reference) Park facility installation (S1), Park renovation/renewal/redesign (S2), Dog park/off-leash areas (S3), Outdoor gym installation/fitness areas (S4), Increasing safe access to parks (S5), Green/natural infrastructure (S6), Physical activity programs (S7), Non-physical activity programs (S8), Playgrounds (S9), Transport to Parks (S10), Use of new pocket parks (S11), and Improve surrounding neighbourhood context (S12). On the other hand, the five different types of demand-side interventions covered in these studies included Park prescription intervention (D1), Education and campaign (D2), Financial incentive (D3), Telecommunication platforms/smartphone technology (D4), Involving community stakeholders (D5) (please refer to the Supplementary Materials for detailed information of interventions).

**Figure 3:**
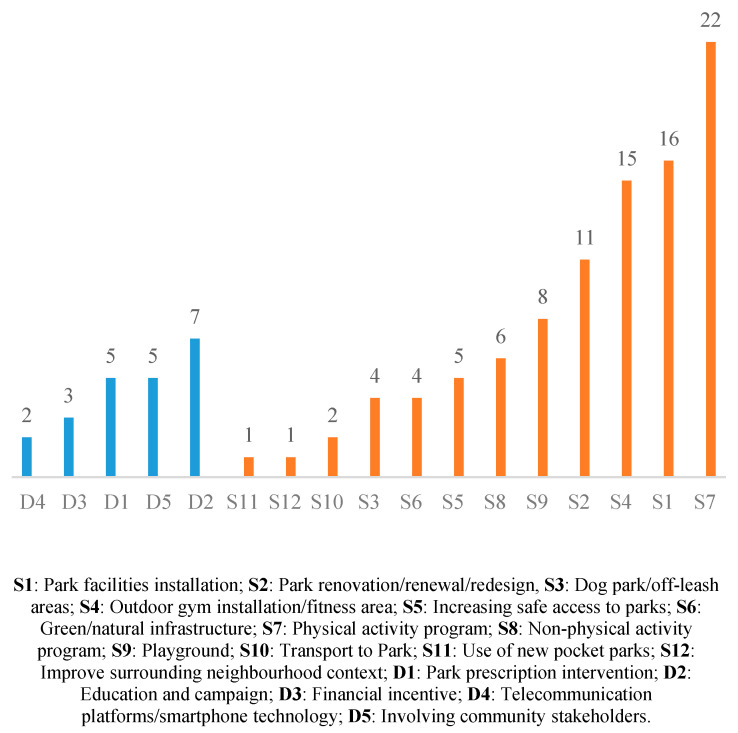
Numbers of studies per intervention that stimulates physical activity in parks. Source: Authors’ own multi-database literature search.

The numbers of studies (by demand and supply) on each type of intervention are reported in Figure 3. Double counting exists as some studies investigated more than one intervention category.

By aggregating the numbers of supply-side and demand-side interventions, there were 95 investigations on supply-side (or 81%) and 22 on demand-side interventions (19%).

**Figure 4:**
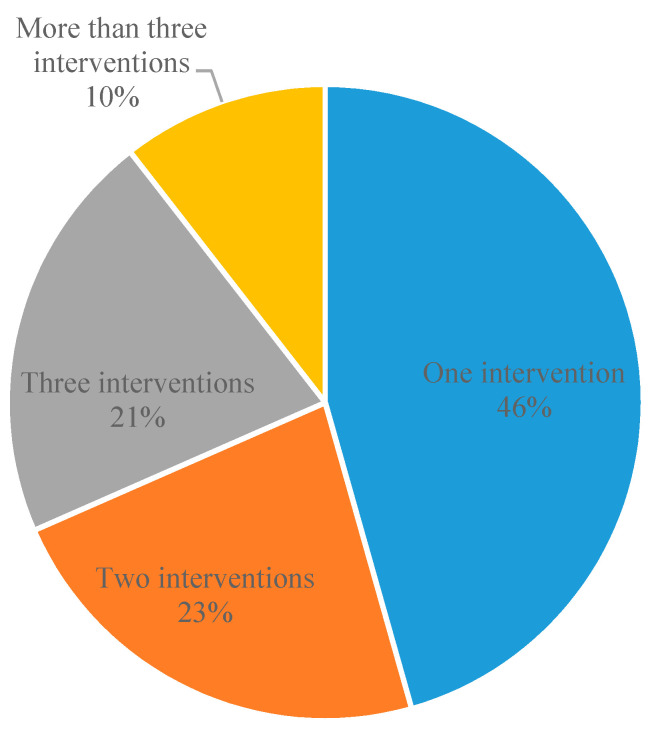
Numbers of interventions assessed in one study. Note: systematic reviews were excluded. Source: Authors’ own multi-database literature search.

Figure 4 shows the number of interventions that are examined in each reviewed study. It is seen that more than half of the selected studies focused on more than one intervention. Understanding this distribution is important as studies simultaneously evaluating multiple interventions might or might not have rigorously addressed potentially confounding effects, and the true impact of one intervention might be incorrectly attributed to another. These concerns generally raise concerns in the current literature review that aims to precisely synthesize the impacts of individual interventions.

Some interventions had been implemented within low-income/high-poverty neighbourhoods as low-income families have lower rates of park use and the lowest level of physical activities [53,54,55,56]. The previous interventions applied to low-income neighbourhoods include renovations within and around parks [57,58,59,60]; physical activity programs in parks [61,62,63]; the park prescription program [43,50,64]; and pocket parks [65]. The renovations within and around parks in low-income neighbourhoods, such as increasing safe access to parks [58]; renovation of outdoor equipment and playfields [57,59]; and improved walkability, incivilities, and aesthetics surrounding parks were found to be associated with greater park use or a significant increase in physical activities in parks.

### 3.5. Outcome Measures and Impacts of Interventions

**Figure 5:**
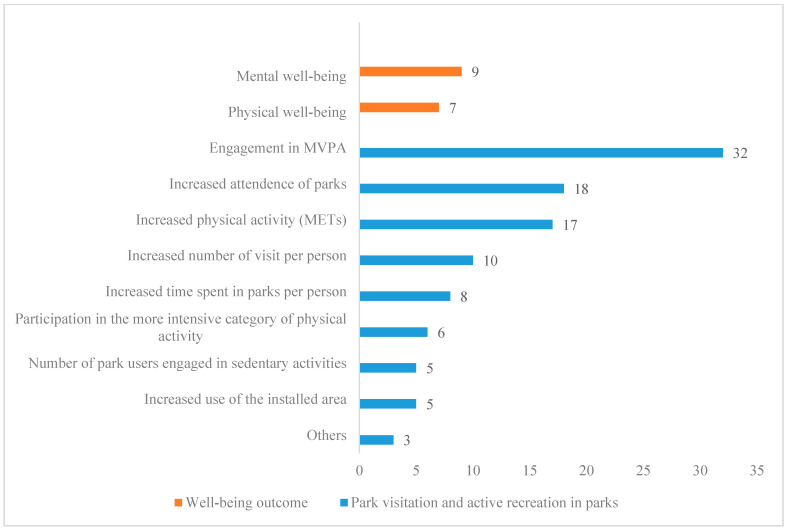
Numbers of studies by outcome measures. Note: Not all studies of well-being outcomes are included; only those measuring active recreation in parks are counted. METs (Metabolic Equivalents) are commonly used to measure the intensity of physical activities. It is the ratio of a person’s working metabolic rate relative to resting metabolic rate. Source: Authors’ own multi-database literature search.

The studies in our identified literature measured the outcome from multiple aspects, using both general well-being measures (physical and mental) and a variety of intervention-specific metrics, including increased use of the installed area; number of sedentary park users; participation in the more intensive category of physical activity; increased time spent in parks per person; increased number of visits per person; increased physical activity as measured by Metabolic Equivalents (METs); increased number of visitors in parks; and engagement in moderate-to-vigorous physical activity (MVPA). Figure 5 reports the number of studies using these measures.

**Figure 6:**
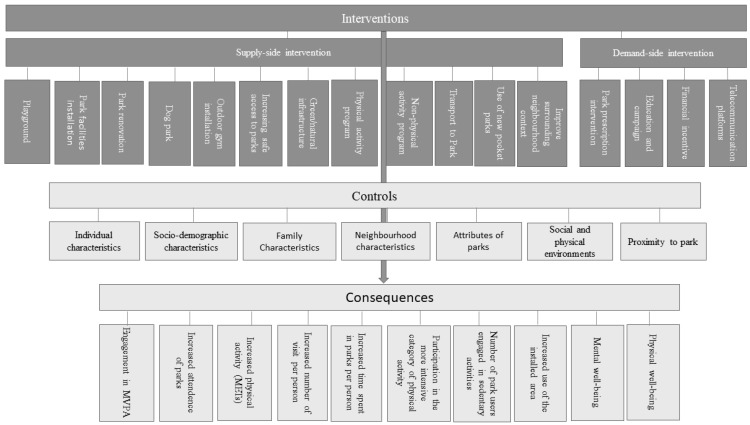
Casual pathways between interventions and park-based physical activities. Source: Authors’ own design.

We attempted to develop a casual pathway analysis of the association between interventions and park use as well as park-based physical activity participation. It consists of three components: (i) identifying the interventions; (ii) identifying the controls that affect the consequences of the interventions; and (iii) identifying the consequences—namely measures of park uses, physical activities, and health outcomes (see Figure 6).

Example studies for supply-side interventions: Playground Park [32], facilities installation [32], Park renovation [66], Dog park [67], Outdoor gym installation [54], Increasing safe access to parks [58], Green/natural infrastructure [68], Physical activity program [69], Non-physical activity program [51], Transport to Park [70], Use of new pocket parks [65], and Improve surrounding neighbourhood context [60]. Example studies for demand-side interventions: Park prescription intervention [43], Education and campaign [71], Financial incentive [61], and Telecommunication platforms [72].

The reviewed studies found mixed results regarding the impacts of interventions in stimulating park use and physical activity participation in parks. In this review, only interventions that were evaluated in at least five publications were summarised, which are education and campaign, park facility installation, park renovation/renewal/redesign, outdoor gym installation in fitness area, physical activity program, non-physical activity program, playground, park prescription intervention, and increasing safe access to parks.

The analytical results are synthesized and reported in Table 4. In the Impact of intervention column (first from left), Mixed findings refer to opposite or contradictory impacts that the intervention was found to have on different outcome measures, while No result means the study had not formally evaluated the intervention outcome but only described its design in the publication.

Table 4 reveals that positive impact is the dominant result of all the selected studies. For the interventions evaluated in at least five publications, about 20–60% of publications reported positive impacts on park use and related physical activity participation. If publications with no results are excluded, the percentages of publications with positive impacts are even higher, ranging from 33% to 100%. Among these, positive impacts are found in all studies (with results provided) regarding park prescription intervention and intervention of increasing safe access to parks. However, the impacts of other interventions are inconclusive in previous studies. The disagreement of the results is due to substantial variability in intervention types, outcome measures, methodologies, as well as analytical procedures used to evaluate the impacts of these interventions (discussed next).

Among these generally mixed results, policy-makers may be interested in those interventions that are found to have positive impacts on physical activity participation in parks. Table S2 in Supplementary Materials reports the studies with positive impacts in a lot more detail as well as highlights the risk of bias in the study.

### 3.6. Risk of Bias

A risk of bias judgment was deemed to be applicable to 44 of the 60 selected publications, which were quantitative with all relevant information properly documented. The evaluation system considers seven potential sources of bias (see the list in the Supplementary Materials) [39,73].

Table 5 reports the itemized and overall bias evaluation results. Heterogeneous ratings are observed among the criteria. While the majority of the studies are considered to have low risk of biases in measurement of interventions and due to departures from intended interventions, a significant proportion are considered to have moderate to serious risks in other five itemized criteria. Common features of those receiving “Serious risk of bias” due to confounding factors include no good method to adjust for the confounding factors or no comparison/control group recruited in the study. Most studies were performed using a non-randomized selection of participants, thereby having a serious risk of bias if no confounding factors were controlled for. Additionally, missing data were common, as many of them had a high attrition rate (a significant proportion of baseline survey respondents were missing in follow-up surveys). In terms of measurement of outcomes, many studies used self-reported outcomes and/or lacked multiple rounds of follow-up surveys and thus faced moderate to serious risks of bias.

Overall, most of the publications (n = 24) suffer from serious bias, accounting for 54.5% of all the selected publications. Publications with critical bias (n = 14) also take up a large proportion, up to 31.8%. However, there are only a few publications with low (n = 1) or moderate bias (n = 5), accounting for 2.3% and 11.4% of the 44 selected publications, respectively.

In contrast, risk of bias in RCT studies (n = 6) is much lower (see Table 6). There is no critical bias in RCT studies, and only 16.7% studies suffer serious bias. Most of RCT studies (n = 4) have moderate bias, which accounts for 66.7%. Moreover, the percentage of RCT studies with low bias (16.7%) is also much higher than that of selected publications (2.3%). Although it is a small sample size for the RCT studies, it indicates that the RCT design could obtain more reliable results and, therefore, are more recommended for park intervention design in the future.

### 3.7. GRADE

As most of the selected studies would suffer from multiple potential risk of bias as discussed above, there might be a lack of credence regarding their empirical findings. Given this notion, it is necessary to further look into the quality of literature findings by outcome.

The quality of evidence regarding several outcome measures that were evaluated in at least five studies was therefore assessed using the “Grading of Recommendations Assessment, Development, and Evaluation” (GRADE) approach [37]. It utilizes a standardized system of rating the quality of publications, incorporating criteria such as the risk of bias, inconsistency of results, indirectness, imprecision, and publication bias (please refer to the Supplementary Materials for detailed grading rules) (In the systematic review, all criteria were discussed by the authors and a consensus was reached regarding the quality evaluation of literature using each of the outcomes).

Table 7 reports the findings. Studies for eight out of nine outcome measures are found to suffer from very serious risk of bias. In addition, most subgroups of literature suffer from inconsistency and publication bias, though indirectness and imprecision are of less concern for the majority of literature. Consequently, the overall GRADE score of each study group by the outcome is consistently low. These results suggest that, while empirical studies have been emerging, there is room for quality improvement to obtain better external validity to assist policy decision-making.

## 4. Discussion

This systematic review aimed to identify the park-based interventions that promote park use and physical activities participation; as well as to evaluate the quality of evidence regarding the effectiveness of these interventions. A systematic approach was applied to identify and screen literature, as well as data extraction. It also used risk of bias judgement and assessment of quality of evidence using the GRADE approach.

The findings showed that studies on park interventions that aimed to stimulate park uses and physical activities in parks are a relatively recent topic, with the first study published in 2009. Nevertheless, since then many different types of interventions have been explored. The interventions were implemented from either the supply side (e.g., provision of parks and access) or the demand side (e.g., stimulation of public participation in park visits and physical activities), with more than eighty percent of studies focussing on supply-side interventions.

In particular, it seems that the demand-side interventions such as *park prescription*, and *education and campaigns* were underutilized (and/or understudied). *Park prescription* interventions are programs designed in collaboration with healthcare providers and prescribe frequency, intensity, time, and type of physical activity for inactive patients. This review found that *park prescription* interventions appeared to be effective (e.g., over 60% of studies of this found positive statistically significant results) in providing knowledge of park locations, nature affinity, and perceived access to parks and therefore promoting park uses and physical activities in parks. However, studies on park prescription interventions were only conducted in United States and Singapore, and there was only a limited number of literature that have studied it. Considering the effectiveness of this intervention, this may be a potential avenue to explore for the future—especially for different cohorts of people who have limited knowledge of the importance of park visitation and physical activities.

The review signalled reasonably strong support for supply-side interventions such as *safe access to parks* (S5), *playgrounds* (S9), and *park renovation and renewal/design* (S2). Despite existing evidence of the association between built environment (i.e., access to public transportation, quality of the environment, sidewalks and biking features, and street characteristics), social environment (i.e., area deprivation and crime-related safety), and park visitations and park-based physical activities [8,16,19,74], there was only limited evidence on the effectiveness of interventions that focused on improvements in the built and social environments of parks. Our review showed the there was only one study on the improvement of surrounding neighbourhood context [60] and two on the intervention of *improving transportation to the park* [32,70]. Richardson et al. [60] showed that park use increased more in the intervention neighbourhood with investment in street characteristics (e.g., improved walkability and aesthetics) than in the comparison neighbourhood. Since the intervention of *improved transport to the park* was used with other interventions [32,70], this then somewhat clouds full knowledge of the effectiveness of this intervention.

There is abundant literature on the interventions applied to low-income neighbour-hoods [57,58,59,60,61,62,63,64,65]. This is because low-income families are documented to have lower rates of park use and the least level of physical activities [53,54,55], which is associated with a lack of park programming, facilities, and amenities, as well as high crime rates and concerns of safety [53,56]. The renovations within and around parks in low-income neighbourhoods [58]; renovation of outdoor equipment and playfields [57,59]; physical activity programs [61,63]; and improved walkability, incivilities, and aesthetics surrounding parks were found to be associated with greater park use or a significant increase in physical activities in parks. Moreover, Razani et al. [43] noticed that increased park use in low-income families was contributed by park prescription, which improved knowledge of park locations, nature affinity, and perceived access to parks. However, Cohen et al. [62] revealed that, because multiple social factors interfere with physical activity among the low-income population, modest interventions such as park programs may not be sufficient to increase park use in low-income neighbourhoods.

The impacts of interventions on park visitation and park-based physical activities were not always consistent among different studies within each intervention, with the exception of the interventions *park* *prescription* and *increasing safe access to parks*. The substantial variability in the outcome measures, study design and analytical processes used to evaluate the impact of environmental interventions is possibly the reason for the inconclusiveness of all results. For example, different study designs such as quasi-experimental evaluation designs with or without a comparison group, randomised controlled trials, and case studies lead to varying robustness of the findings. Randomised controlled trials are considered one of the best methods to reduce certain sources of bias, while case studies usually lead to the least reliable results. There is also the opportunity for more quasi-experimental statistical approaches such as difference-in-difference to be applied in the future to increase results reliability and generalizability.

Our quality assessment showed that over 80% of selected studies had critical or serious overall bias, and all studies within each outcome suffered very low quality of evidence using the GRADE approach. First, few studies used appropriate analysis methods to adjust confounders. While individual characteristics such as gender and age were commonly controlled in the studies analysis, other confounders such as socioeconomic characteristics of individuals and communities were only controlled for in some studies. Second, most studies suffered from serious selection bias and were frequently limited by a lack of representativeness. Third, missing data were common in selected studies, as many of them had a high attrition rate. Moreover, many studies lacked multiple rounds of follow-up surveys or used self-reported outcomes, which were not reliable. In addition to risk of bias, most studies suffered from inconsistency as well as imprecision, which leads to low quality of evidence and very low GRADE scores.

Some limitations of this review include the fact that it only accessed English-published studies. Due to language constraints, it might potentially affect our analysis in non-English speaking countries, while we might undercount research conducted in these countries. Future studies need to fill this gap by investigating the possible effect of these studies on the findings.

Additionally, all results suggest that there is a need for more methodologically sound study designs on park interventions with higher study quality and reduced bias. These include the use of randomized controlled trials, better matching of control sites and use of multiple control sites, employment of strategies to improve response rate, appropriate methods to control the confounders, the use of quasi-experimental methods, the use of adequate outcome measurements, and measuring exposure to the intervention at the individual level. Moreover, most studies investigated the impacts of multiple interventions, thereby limiting the identification of the specific effect of each intervention. We suggest that future evaluation design should focus on one specific intervention at a time to isolate the effect of this intervention. Since there is a lack of research specific to older people and children, there is a need to further explore the impact of park-based interventions on older people and children. Additionally, more future studies need to investigate whether physical features (e.g., renovation of exercise machines) or social factors (e.g., socializing and outdoor activities) play a more critical role in attracting older people to parks.

Finally, given the association between social and built environments on park-based physical activities, future studies are needed to understand the effectiveness of interventions to improve the surrounding neighbourhood context or to change the social and built environment. Due to a lack of evidence, the impacts of other interventions such as the use of new pocket parks, telecommunication platforms/smartphone technology, and transport to the park also need to be further investigated. There is also a call for wider nature experiments—such as the impact of ‘greening suburbs’ and increasing tree cover on the impact of the health, wellbeing, and environmental outcomes of local communities in the future.

## 5. Conclusions

This systematic review identified and coded 44 articles that focused on interventions to promote park use and physical activity participation in parks and the effectiveness of these interventions. Specifically, the findings of this systematic review show promise in increasing park visitation and physical activity participation in parks. Possible interventions that promote park visitation and physical activities can be broken down into demand and supply-based interventions.

Supply-based interventions included park facility installation, park renovation/renewal/redesign, dog park/off-leash areas, outdoor gym installation/fitness areas, increasing safe access to parks, green/natural infrastructure, physical activity program, non-physical activity programs, playgrounds, transport to park, use of new pocket parks, and improvement of surrounding neighbourhood context. Demand-based interventions included park prescription interventions, education, campaigns, financial incentives, telecommunication platforms/smartphone technology, and involving community stakeholders in parks and management.

The strongest evidence was found for park prescriptions; safe access to parks; playgrounds, and park renovation and renewal/design. However, there seemed to be inconsistency in many findings due to the low quality of evidence and serious results bias. The benefits of many interventions would require further investigation. Specifically, randomized controlled trials or high-quality natural experiments may need to be designed in future studies.

## Figures and Tables

**Table 1:** Geographical coverage of selected peer-reviewed publications.

Country/Region	Number of Studies
USA	33
Australia	16
Canada	3
Singapore	2
Colombia	2
Japan	1
Netherlands	1
Denmark	1
Italy	1
Belgium	1
England	1
Scotland	1
New Zealand	1
Total	64

Source: Authors’ own multi-database literature search.

**Table 2:** Study design of selected publications.

Study Design	Number of Studies
Randomised controlled trial	12
Quasi-experimental pre–post evaluation design with a comparison group	27
Quasi-experimental pre–post evaluation design without a parallel comparison group	14
Case study	3
Systematic reviews	4

Source: Authors’ own multi-database literature search.

**Table 3:** Population of interest in selected publications.

Population of Interest	Number of Studies
Whole population	28
Children	6
Adults	7
Low-income families	12
Older people	2
Women	2
Adolescents	2
Youths	1

Note: More than one population group could be studied within one publication.

**Table 4:** Summary of empirical evaluated impacts of interventions.

Impact of Intervention	Park Prescription Intervention–D1	Increasing Safe Access to Parks–S5	Play-Grounds–S9	Park Renovation/Renewal/Redesign–S2	Education and Campaign –D2	Physical Activity Program–S7	Park Facilities Install-ation-S1	Non-Physical Activity Program–S8	Outdoor Gym Installation/Fitness Area–S4	Involving Community Stakeholders–D5
Positively statistically significant	3 (60%)	3 (60%)	4 (50%)	5 (46%)	3 (43%)	9 (41%)	6 (38%)	2 (34%)	4 (27%)	1 (20%)
Negatively statistically significant	0 (0%)	0 (0%)	1 (13%)	0 (0%)	1 (14%)	0 (0%)	2 (12%)	0 (0%)	1 (7%)	0 (0%)
Mixed	0	0	0 (0%)	2 (18%)	1 (14%)	3 (14%)	2 (13%)	2 (33%)	3 (20%)	1 (20%)
Not significant	0	0	1 (12%)	0 (0%)	0 (0%)	3 (14%)	2 (12%)	0 (0%)	3 (20%)	1 (20%)
No result provided	2 (40%)	2 (40%)	2 (25%)	4 (36%)	2 (29%)	7 (31%)	4 (25%)	2 (33%)	4 (26%)	2 (40%)
Total	5	5	8	11	7	22	16	6	15	5

Note: Only interventions with at least five studies are included in this table. Percentages are in parenthesis. Source: Authors’ own multi-database literature search and synthesis.

**Table 5:** Risk of bias judgment outcomes of all selected studies (n = 44).

Risk of Bias Judgement	Critical	Serious	Moderate	Low
Bias due to confounding effects	2	27	10	5
Bias in selection of study participants	2	30	4	8
Bias in measurement of interventions	0	7	9	28
Bias due to departures from intended interventions	0	0	0	44
Bias due to missing data	1	31	4	8
Bias in measurement of outcomes	0	16	20	8
Bias in selection of the reported results	0	0	25	19
Overall bias	14	24	5	1

Source: Authors’ own multi-database literature search and synthesis.

**Table 6:** Risk of bias judgment outcomes of the RCT studies (n = 6).

Risk of Bias Judgement	Critical	Serious	Moderate	Low
Bias due to confounding effects	0	1	2	3
Bias in selection of study participants	0	0	1	5
Bias in measurement of interventions	0	0	0	6
Bias due to departures from intended interventions	0	0	0	6
Bias due to missing data	0	1	2	3
Bias in measurement of outcomes	0	1	2	3
Bias in selection of the reported results	0	0	1	5
Overall bias	0	1	4	1

Source: Authors’ own multi-database literature search and synthesis. Note: The RCT studies in this table are RCT studies with results provided.

**Table 7:** Assessment of quality of studies using the GRADE approach.

Outcome Number of Studies	Risk of Bias	Inconsistency	Indirectness	Imprecision	Publication Bias	Overall GRADE Score
Engagement in MVPA (32)	Very serious (−2)	Serious (−1)	Serious (−1)	Serious (−1)	Unlikely	Very low
Increased attendance of parks (18)	Very serious (−2)	Serious (−1)	No important indirectness	Serious (−1)	Likely (−1)	Very low
Increased physical activity (METs) (17)	Very serious (−2)	Serious (−1)	No important indirectness	No important imprecision	Likely (−1)	Very low
Increased number of visit per person (10)	Very serious (−2)	No important inconsistency	No important indirectness	No important imprecision	Likely (−1)	Very low
Increased time spent in parks per person (8)	Very serious (−2)	Very serious (−2)	No important indirectness	No important imprecision	Unlikely	Very low
Participation in the more intensive category of physical activity (6)	Very serious (−2)	Serious (−1)	No important indirectness	No important imprecision	Likely (−1)	Very low
The number of physically inactive visitors (5)	Serious (−1)	Serious (−1)	Serious (−1)	No important imprecision	Unlikely	Very low
Increased use of the installed area (5)	Very serious (−2)	No important inconsistency	No important indirectness	Serious (−1)	Likely (−1)	Very low

Source: Authors’ own multi-database literature search and synthesis.

## Data Availability

All the data used in the conduction of this review are available from the corresponding author upon reasonable request.

## Supplementary Materials

The following supporting information can be downloaded at https://www.mdpi.com/article/10.3390/ijerph191912590/s1, Supplement File S1: Interventions covered in this study; Supplement File S2: Risk of bias; Supplement File S3: GRADE; Supplement File S4: Tables S1–S3. References [75,76,77,78,79,80,81,82,83,84] are cited in Supplementary Materials.
