# Supplementary material for: The Effectiveness of Interventions to Increase Participation and Physical Activities in Parks: A Systematic Review of the Literature
Source: Int J Environ Res Public Health. 2022 Oct 2;19(19):12590. doi: 10.3390/ijerph191912590 (PMC9566024; doi:10.3390/ijerph191912590)
Supplement: Supplementary file 1 [file ijerph-19-12590-s001.zip › ijerph-1875773-supplementary.pdf]

International Journal of
Environmental Research and Public Health

MDPI

Supplementary Materials

Supplement File S1: Interventions covered in this study

12 supply-side interventions covered in this study included (numbered for later reference):

- *Park facility installation* (S1): park facility installation including all the installation/construction of park facilities (e.g. BBQ stations, shade sails, lighting, and picnic benches) except the installation of physical activities related facilities such as gym/fitness area or playground.
- *Park renovation/renewal/redesign* (S2): park renovation/renewal/redesign including the renewal or renovation of all types of park facilities (physical activities related facilities such as gym/fitness area or playground are included).
- *Dog park/off-leash areas* (S3): park/area that is designed specifically for dog activities and has off-leash permission.
- *Outdoor gym installation/fitness areas* (S4): outdoor exercise equipment, fitness equipment, or walking and cycle tracks that are specifically constructed for adults.
- *Increasing safe access to parks* (S5): increase safe access to parks or reduce the barriers to access the parks (e.g. installation of a signalized crosswalk, prevent motor vehicle access to parks).
- *Green/natural infrastructure* (S6): adding new green space or higher tree cover.
- *Physical activity program*s (S7): providing human hours of group exercise class (e.g. dance classes) and physical activity programs (e.g. walking programs, family soccer) in parks.
- *Non-physical activity programs* (S8): non-physical activity programs or social events held in parks (e.g. seminars on the adoption of a healthy lifestyle, cooking demos, coffee talks).
- *Playgrounds* (S9): children's playground installation or renovation in parks.
- *Transport to Parks* (S10): providing adequate active transport infrastructure to parks.
- *Use of new pocket parks* (S11): small parks (less than one acre) which generally serve the immediate population living within one quarter to one-half mile.
- Improve surrounding neighbourhood context (S12): renovation and the changing environment surrounding the parks (e.g. investment on street characteristics such as walkability, amenities, incivilities, and poor aesthetics).

5 different types of demand-side interventions covered in these studies included:

- Park prescription intervention (D1): The prescription provided by medical professionals, which usually includes the importance of engaging in physical activities, the possibility of engaging in physical activity in a park, and information on frequency, intensity, time, and location of activities
- Education and campaign (D2): education and campaign for physical activities by providing the importance of the physical activities or the opportunities of physical activity programs in parks
- Financial incentive (D3): prizes given to participants based on their physical activities in park or park visitations (e.g, healthy mileage reward program, prize based on number of park visitations)
- Telecommunication platforms/smartphone technology (D4): smartphone technology or telecommunication platforms used to promote physical activities in parks (e.g. ecofit app which includes workout plans tailored to different locations, difficulty levels, and workout types)
- Involving community stakeholders (D5): involving community members and/or stakeholders in the form of park advisory boards (PABs) which play a role in planning and programming to increase park use and park-based physical activity.

Supplement File S2: Risk of bias

According to relevant studies of risk of bias [39,73], the bias sources of evaluation system including,

1. Confounding effects:
a. Was there an appropriate analysis method to adjust for potential confounding effects?
b. Were there any differences in baseline demographic characteristics?
c. Was the control site well matched to the intervention site?
d. Were there multiple control sites?
2. Selection of participants into the study:
a. Were there systematic differences between intervention and control groups?
b. Was there a clear explanation of sample?
3. Measurement of interventions:
a. Did the study explain what was modified in the intervention?
b. Where was the intervention implemented?
c. How long did it ttake to construct the intervention?
4. Departures from intended interventions:
a. Did the study rigorously measure intervention exposure?
5. Missing data:
a. Was there a missing data issue?
b. How severe was the issue?
6. Measurement of outcomes:

a. Was the outcome measure reliable?
b. Were the outcomes measured over a period of more than one week at each time point
c. Were there multiple follow-up time points?
7. Selection of the reported results:
o Was a study potocol published?

Each criterion is evaluated as having a risk level that is critical, serious, moderate or low. The lower the risk level is, the more reliable the study is considered to be. There is an overall bias evaluation following these itemized criteria. A more detailed explanation of the risk of bias judgment method is presented in Table S3.

Supplement File S3: GRADE

The detailed rules of the GRADE approach are as follows,

- First, an initial quality score is assigned. If a study is randomized controlled trials, the initial quality is considered high and four points are added (+4). Alternatively, if it is an observational study, the initial quality is low and only two points are added (+2).
- Risk of bias assessment rule: one point is deducted if the study fails to adequately control for confounding (-1), or completely account for outcome events (-1), or have a sample size below 100 (-1), or completely report the outcomes (-1). If a study fails all four criteria, four points are taken from the initial score.
- Inconsistency assessment rule: one point is taken (-1) from the initial score if the study either presents no subgroup analysis or if it fails to use heteroskedasticity robust standard errors. If a study has both issues, two points are taken.
- Indirectness assessment rule: one point is taken (-1) from the initial score if study if the study focuses on a different (sub)population other than that of interest, or if the intervention tested differs from the intervention of interest, or if the outcomes differ from those of primary interest. If a study suffers from all three issues, three points are taken.
- Imprecision assessment rule: one point is taken (-1) if the hypothesized impact is significant at 5% rather than 1% level; two points are taken (-2) if it only significant at 10% or not significant.
- For each outcome that has been assessed by more than five publications, the averages of these above criteria are taken and rounded to the nearest integer.
- Publication bias assessment rule: for the several publications that evaluate the impact of the same intervention on the same outcome, one point is taken (-1) if all studies present impact estimates that are have the same sign. On the contrary, no point is taken if both positive and negative estimated impacts are reported across these studies.
- Finally, an overall GRADE score for the subgroup of literature focusing on each outcome is algebraically calculated following the above procedure. The overall GRADE score takes one of the following value: high (if the score is +4), moderate (+3), low (+2) or very low (+1).

Supplement File S4: Tables

Table S1. Searching strings used to identify interventions to facilitate park active recreation

Data-base	Searching String
**PubMed**	("park"[Title/Abstract] OR "reservoir" OR "botanic garden" OR "forestry reserves")[Title/Abstract] AND ("physical activity"OR"visit"OR"visitation"OR" walk"OR"cycling"OR"bike"OR"biking"OR"participation"OR"cycle"OR"exercise"OR"play"OR"recreation"OR"leisure"OR"sport"OR"outdoor activity") [Title/Abstract] AND ("intervention" OR "randomised control" OR "randomised control" OR " control group" OR "experiment")[Title/Abstract]
**Web of Science**	TS=(("park" OR "reservoir" OR "botanic garden" OR "forestry reserves") AND ("physical activity" OR "visit" OR "visitation" OR "walk" OR "cycling" OR "bike" OR "biking" OR “participation” OR "cycle" OR "exercise" OR "play" OR "recreation" OR "leisure" OR "sport" OR "outdoor activity") AND ("intervention" OR "randomised control" OR "randomised control" OR "control group" OR "experiment" OR "control group"))
**Scopus**	TITLE-ABS-KEY (("park" OR "reservoir" OR "botanic garden" OR "forestry reserves") AND ("physical activity" OR "visit" OR "visitation" OR "walk" OR "cycling" OR "bike" OR "biking" OR “participation” OR "cycle" OR "exercise" OR "play" OR "recreation" OR "leisure" OR "sport" OR "outdoor activity") AND ("intervention" OR "randomised control" OR "randomised control" OR "experiment" OR "control group"))
**CINAHL**	AB (("park" OR "reservoir" OR "botanic garden" OR "forestry reserves") AND ("physical activity"OR"visit"OR"visitation"OR"walk"OR"cycling"OR"bike"OR"biking"OR”participation”OR"cycle"OR"exercise"OR"play"OR"recreation"OR"leisure"OR"sport"OR"outdoor activity") AND ("intervention" OR "randomised control" OR "randomised control" OR "comparative study" OR "control group" OR "experiment" OR "program" OR "pre-test" OR "post-

test")) OR TI (("park" OR "reservoir" OR "botanic garden" OR "forestry reserves") AND ("physical activity"OR"visit"OR"visitation"OR"walk"OR"cycling"OR"bike"OR"biking"OR"participation"OR"cycle"OR"exercise"OR"play"OR"recreation"OR"leisure"OR"sport"OR"outdoor activity") AND ("intervention" OR "randomised control" OR "randomised control" OR "comparative study" OR "control group" OR "experiment" OR "program" OR "pre-test" OR "post-test"))

Source: authors' own design.

Table S2. Summary of studies by intervention finding statistically significant positive impacts on park visitation and active recreation in parks (n=33)

Intervention type	Study	Details of intervention	Main finding	Overall risk of bias
D2: Education and campaign	Hoehner et al. [75]	Encourage park or trail use. Three parks used park rangers, and others used community events and programming. One park launched a walking program with team competition, ranger-le hikes.	Five of the 7 projects showed evidence of an increase in physical activity that was associated with the intervention activities.	Critical
	Kaczynski et al. [76]	White text on the sign read, "Take a walk around the park! Doctors recommend that being active just 30 minutes per day can help you maintain a healthy weight and ward off many diseases."	Participants who were exposed to the park photo with the sign reported significantly greater intentions to be active than those who viewed the photo without a sign.	Serious
	Sharpe et al. [77]	24-week behavioural intervention and media campaign	Women in the behavioural intervention had statistically significant positive changes on park use and physical activity minutes.	Critical
S1: Park facility installation	Buller et al. [78]	Construction of shade sails	The results showed that building shade sails in public parks increased the likelihood that adults would choose to use shaded PRAs more than unshaded ones.	Moderate
	Dobbinson [66]	Two of the intervention parks received new playground equipment with a shade sail and new walking paths. The other intervention park gained only minor playground amenities.	The study found more visitors used the refurbished parks and increased mean number of park visitors engaging in moderate-to-vigorous physical activity.	Critical
	Lal et al. [79]	Installation of a play-scape located in a low socio-economic area	The new play-scape resulted in an overall incremental net gain of 114,114 MET-h compared with the control park.	Critical
	Smith et al. [32]	Multiple interventions; literature review	Findings showed a positive effect of walkability components, provision of quality parks and playgrounds, and installation of or improvements in active transport infrastructure on active transport, physical activity, and visits or use of settings.	
	Tester and Baker [59]	In both parks, artificial turf replaced uneven dirt fields, and new fencing, landscaping, lighting, and picnic benches were added. In Park A, permanent soccer goals were installed, and in Park B, a walkway around the field was restored.	Both intervention park playfields saw significant increases in park visitation and a significant increase in sedentary, moderately active, and vigorously active visitors to the intervention park playfields.	Serious
	Veitch et al. (2012) [80]	Establishment of a fenced leash-free area for dogs; an all-abilities playground; a 365-m walking track; a barbecue area; landscaping; and fencing, to prevent motor vehicle access to the park.	There were significant increases from pre- to post-improvement in the number of park users and the number of people observed walking and being vigorously active in intervention park, relative to control park.	Serious
S2: Park renovation/renewal/redesign	Dobbinson [66]	Two of the intervention parks received new playground equipment with a shade sail and new walking paths. The other intervention park gained only minor playground amenities.	The study found more visitors used the refurbished parks and increased mean number of park visitors engaging in moderate-to-vigorous physical activity.	Critical

	Roemmich et al. [81]	Manipulation of location of seating around a park playground (A: Usual seating arrangement; B: Seating removed)	For adults, the odds of being in moderate-to-vigorous physical activity than sitting were greater, and MET intensities were greater. The duration families stayed at the park did not differ across conditions.	Critical
	Slater et al. [82]	Involvement of community groups in playground design selection, installation, and ongoing maintenance	Significant increases between baseline and 12-month follow-up were found for park utilization and the number of people engaged in MVPA.	Serious
	Smith et al. [32]	Multiple interventions; literature review	Findings showed a positive effect of walkability components, provision of quality parks and playgrounds, and installation of or improvements in active transport infrastructure on active transport, physical activity, and visits or use of settings.	
	Veitch et al. [80]	Establishment of a fenced leash-free area for dogs; an all-abilities playground; a 365-m walking track; a barbecue area; landscaping; and fencing, to prevent motor vehicle access to the park.	There were significant increases from pre- to post-improvement in the number of park users and the number of people observed walking and being vigorously active in intervention park, relative to control park.	Serious
S4: Outdoor gym installation/fitness area	Copeland et al. [46]	In 1 active park 5 machines were clustered together and the second active park had 5 separate stations, each with 3 to 4 machines and walking paths connecting them.	The number of adults that visited the regular parks was significantly less than active parks. Estimated energy expenditure was also significantly higher among adults observed in active parks than regular parks.	Critical
	Dobbinson [66]	Two of the intervention parks received new playground equipment with a shade sail and new walking paths. The other intervention park gained only minor playground amenities.	The study found more visitors used the refurbished parks and increased mean number of park visitors engaging in moderate-to-vigorous physical activity.	Critical
	Sami et al. [83]	Install 8 pieces of equipment in fitness zone	MET score at post intervention is higher than that of pre-intervention. Post-intervention users in the park had higher odds of being classified in a more active category than were pre-intervention users.	Serious
	Sami et al. [84]	Adding outdoor exercise equipment (OEE) in two parks in Garden Grove and Anaheim	MET score at post-intervention is higher than that of pre-intervention. Post-intervention users in the park had higher odds of being classified in a more active category than were pre-intervention users.	Serious
S7: Physical activity program	Balcázar et al. [45]	Lifestyle exercise and environmental exercise; lifestyle nutrition and environmental nutrition	The intervention was successful in promoting use of recreational facilities among border residents at high risk for cardiovascular disease.	Critical
	Banda et al. [71]	Event activities included group walks, child and adult activities and games, and music via a radio station truck. A pedometer, map of county walking tracks and trails	Park use was significantly greater at baseline than post campaign. There were no significant differences in the number of park users engaged in sedentary, walking, and vigorous activities between baseline and post campaign.	Serious
	Gagliardi [51]	Organization of socializing activities e.g., seminars; outdoor activities	The quantitative results showed that the participants' level of physical activity increased significantly.	Critical
	Han et al. [61]	The quantitative results showed that the participants' level of physical activity increased significantly.	The study park had a higher number of parks users and METs than other parks with similar park conditions and neighbourhood characteristics.	Serious
	Hoehner et al [75]	Encourage park or trail use. Three parks used park rangers, and others used community events and programming. One park launched a walking program with team competition, ranger-le hikes.	Five of the 7 projects showed evidence of an increase in physical activity that was associated with the intervention activities.	Critical
	Müller-Riemenschneider et	1) Face-to-face Park Prescription; 2) invitation to weekly exercise sessions in parks.	Mean minutes of MVPA per week (primary outcome) was greater in the PPI than the control group.	Moderate

	al. [49]			
	Razani et al. [43]	Each family met with a paediatrician. Families were randomized into two groups: a supported group was invited to three organized group outings to parks, and the other group was free to visit parks on their own.	Park use increased as participants reported increased level of information about the location of parks, nature affinity and perceptions about time and resource availability.	Moderate
	Razani et al. [50]	Parents in both groups received physician counselling. The supported group attended three weekly family nature outings.	Over the three month course of study, Park visits increased significantly.	Low
	Torres et al. [63]	Implementing new Recreovias in parks	Users from existing Recreovias were significantly more active and significantly higher vigorous activity.	Serious
S8: Non-physical activity program	Balcázar et al. [45]	Lifestyle exercise and environmental exercise; lifestyle nutrition and environmental nutrition	The intervention was successful in promoting use of recreational facilities among border residents at high risk for cardiovascular disease	Critical
	Gagliardi [51]	Organization of socializing activities e.g., seminars; outdoor activities	The quantitative results showed that the participants' level of physical activity increased significantly.	Critical
S9: Playground	Slater et al. [82]	Involvement of community groups in playground design selection, installation, and ongoing maintenance	Significant increases between baseline and 12-month follow-up were found for park utilization and the number of people engaged in MVPA.	Serious
	Smith et al. [32]	Multiple interventions; literature review	Findings showed a positive effect of walkability components, provision of quality parks and playgrounds, and installation of or improvements in active transport infrastructure on active transport, physical activity, and visits or use of settings.	
	Veitch et al. [80]	Establishment of a fenced leash-free area for dogs; an all-abilities playground; a 365-m walking track; a barbecue area; landscaping; and fencing, to prevent motor vehicle access to the park.	There were significant increases from pre- to post-improvement in the number of park users and the number of people observed walking and being vigorously active in intervention park, relative to control park.	Serious
	Veitch et al. [12]	The new equipment included a large 360-degree swing, traditional swing set, maze, rockers, sandpit, nature play area, climbing equipment, landscaping, and various sculptures and was designed to be accessible for children with disabilities.	The observational data showed increase in park visitor and counts of visitors engaged in MVPA at the intervention park relative to the control park.	Serious

Source: Authors' own multi-database literature search and synthesis.

Table S3. Summary of the seven domains and types of signaling questions added to the ACROBAT-NRSI

Bias domain	Definition	Signaling questions
Bias due to confounding	Confounding occurs when one or more variables also explain the observed relationship between exposure and outcome.	Did the authors use an appropriate analysis method that adjusted for all the critically important confounding domains?<br>Critically important confounding domains not controlled for<br>Differences in baseline outcome measurements<br>Differences in baseline demographic characteristics<br>Any unusual events<br>Socioeconomic or political influences<br>What variables were used to match intervention and control sites?<br>Demographic variables (e.g. ethnicity, income, education)<br>Features, factilites, or amenities<br>Sizes<br>Land use<br>Same neighborhood<br>Is the control site matched to the intervention site?<br>Were there multiple control sites?

Bias in selection of participants into the study	This bias domain refers to the exclusion of eligible participants that biases the outcome.	Is there a fully justified sample size calculation?<br>Is there a clear and sufficient description of the sample?
Bias in measurement of interventions	Bias in this domain occurs when intervention status is misclassified; that is, when errors in measuring participants exposure to the intervention biases the estimated effect of the intervention.	Did the authors describe…<br>…what was modified in the intervention?<br>…where the intervention was implemented?<br>…how long it took to construct the intervention?
Bias due to departures from intended interventions	This bias domain refers to systematic differences between intervention and control groups due to departures from the intended intervention.	Was individual-level intervention exposure measured?<br>Was individual-level intervention exposure measured objectively?
Bias due to missing data	Studies that have missing data increase the risk of selection bias, thus resulting in a misrepresented sample.	Response rate at baseline, follow-up, and the overall response rate.
Bias in measurement of outcomes	Bias can occur when there are errors in measuring the outcomes of the intervention.	Was the outcome measure valid and reliable?<br>Were the outcomes measured over a period of more than one week at each time point?<br>Were there multiple follow-up time points?
Bias in selection of the reported result	This domain refers to the selective reporting of fully reported results.	Was a study protocol published?<br>Did the authors provide a clear and compelling justification for not publishing a study protocol?

Sources: Sterne et al. [39] and Benton et al. [73].
